# PAC-Net: Multi-pathway FPN with position attention guided connections and vertex distance IoU for 3D medical image detection

**DOI:** 10.3389/fbioe.2023.1049555

**Published:** 2023-02-02

**Authors:** Zhenghua Xu, Tianrun Li, Yunxin Liu, Yuefu Zhan, Junyang Chen, Thomas Lukasiewicz

**Affiliations:** ^1^ State Key Laboratory of Reliability and Intelligence of Electrical Equipment, School of Health Sciences and Biomedical Engineering, Hebei University of Technology, Tianjin, China; ^2^ Department of Radiology, Hainan Women and Children’s Medical Center, Haikou, China; ^3^ College of Computer Science and Software Engineering, Guangdong Laboratory of Artificial Intelligence and Digital Economy (SZ), Shenzhen University, Shenzhen, China; ^4^ Department of Computer Science, University of Oxford, Oxford, United Kingdom

**Keywords:** computer-aided diagnosis, 3D medical image detection, position attention guided connection, multi-pathway FPN, vertex distance IoU

## Abstract

Automatic medical image detection aims to utilize artificial intelligence techniques to detect lesions in medical images accurately and efficiently, which is one of the most important tasks in computer-aided diagnosis (CAD) systems, and can be embedded into portable imaging devices for intelligent Point of Care (PoC) Diagnostics. The Feature Pyramid Networks (FPN) based models are widely used deep-learning-based solutions for automatic medical image detection. However, FPN-based medical lesion detection models have two shortcomings: the object position offset problem and the degradation problem of IoU-based loss. Therefore, in this work, we propose a novel FPN-based backbone model, i.e., Multi-Pathway Feature Pyramid Networks with Position Attention Guided Connections and Vertex Distance IoU (abbreviated as PAC-Net), to replace vanilla FPN for more accurate lesion detection, where two innovative improvements, a position attention guided connection (PAC) module and Vertex Distance IoU Vertex Distance Intersection over Union loss, are proposed to address the above-mentioned shortcomings of vanilla FPN, respectively. Extensive experiments are conducted on a public medical image detection dataset, i.e., Deeplesion, and the results showed that i) PAC-Net outperforms all state-of-the-art FPN-based depth models in both evaluation metrics of lesion detection on the DeepLesion dataset, ii) the proposed PAC module and VDIoU loss are both effective and important for PAC-Net to achieve a superior performance in automatic medical image detection tasks, and iii) the proposed VDIoU loss converges more quickly than the existing IoU-based losses, making PAC-Net an accurate and also highly efficient 3D medical image detection model.

## 1 Introduction

Automatic medical image detection aims to utilize artificial intelligence techniques to detect lesions in medical images accurately and efficiently, which is one of the most important tasks in computer-aided diagnosis (CAD) systems, and can be embedded into portable imaging devices for intelligent Point of Care (PoC) Diagnostics (Manhas et al., 2021). Recently, with the continuous development of artificial intelligence, deep-learning-based methods have started to play an important part in automatic medical image detection (Wang et al., 2018; Wang et al., 2017; Lee et al., 2018). Deep-learning-based detection models that utilize convolutional neural networks (CNNs) to learn the features of input images, such as vanilla Faster R-CNN (Ren et al., 2015) and vanilla YOLO (Redmon et al., 2016), have been increasingly adopted in medical image detection tasks.

However, the detection of medical images is often more difficult than detecting objects within natural images because of two reasons: i) the similarity between the object and the background is higher in medical images than that in natural images, which increases the difficulty of object detection (Greenspan et al., 2016), and ii) medical images often contain small but important lesions and organs as the detection objects, whose detailed information may be lost in the deep convolutional processing for feature learning (Ren et al., 2018). Therefore, to learn features better, medical image detection works based on models such as Faster R-CNN and YOLO (Li et al., 2019; Tang et al., 2019; Zhao et al., 2019; Zlocha et al., 2019) have basically introduced the Feature Pyramid Networks (FPN) (Lin et al., 2017a) to replace the traditional CNN as the feature learning backbone. Specifically, differently from conventional CNNs, FPN first uses a bottom-up path to extract features by convolution, then adds a top-down pathway to upsample the high-level features, and then combines them with the corresponding previous layer features by lateral concatenation. By using FPN as the backbone, the advanced detection models can fuse features of different scales and depths in the process of feature learning, making the learned feature information richer and more complete and avoiding the loss of detailed information (He et al., 2017; Liu et al., 2018). For example (Zlocha et al., 2019), uses FPN as the backbone of the retina detection model to achieve the detection of lesions in CT images. Similarly (Zhao et al., 2019), proposes an FPN-based PFA-ScanNet for the detection of breast cancer. Further, to fully utilize the rich spatial contextual information in 3D medical datasets (Li et al., 2019), proposes an FPN-based MVP-Net using a three-pathway input to help the network learn the features of spatial contextual information in 3D CT images and achieved good detection results.

However, the existing detection models mainly suffer from two shortcomings: i) Object position offset problem (Lin et al., 2017a): To achieve the fusion of multi-scale features, FPN needs to upsample the features of each layer of the top-down pyramid and then fill the expanded pixels with the nearest neighbor interpolation. This will inevitably cause the offset of the object’s position. Although FPN fuses features with accurate position information bottom-up by lateral connections to remedy the problem of position offset, the simple lateral connections do not completely solve the problem. Therefore, FPN still has the problem of position offset. However, the position information of the object is very important for detection. When the position of the detection object is shifted in the feature map, it will inevitably make the region proposals (RPs) selected by RPN deviate from the object’s actual position in the input image to a certain extent, thus causing the inaccuracy of detection. ii) Degradation problem of IoU-based loss (Zheng et al., 2020): Most of the existing works use the original Iou loss (Yu et al., 2016) or IoU-based loss function, such as GIoU (Rezatofighi et al., 2019) and DIoU (Zheng et al., 2020). But most of them have certain problems, such as the IoU loss only judges the bbox quality based on IoU thus leading to giving the same loss in the face of RPs with different quality. Although GIoU or DIoU take other factors into account, when there exists a mutual inclusion of RPs and ground truth (GT) boxes, or there exists some RPs with the same distance from the center point of GT, the calculation of GIoU and DIoU will degrade to ordinary IoU loss, thus wrongly getting the same IoU loss value on bboxes of different quality, which affects the speed and accuracy of model training.

Therefore, in this work, we propose a novel Multi-Pathway FPN with Position Attention Guided Connections and Vertex Distance IoU model, named PAC-Net, for 3D medical image detection. Generally, PAC-Net addresses the above two problems by first proposing a new position attention guided connection module to remedy the object position offset problem caused by upsampling operations on FPN and then proposing a novel Vertex Distance IoU (VDIoU) loss to solve the existing problem of inaccurate calculation of IoU-based losses. Specifically, we first add position attention guided blocks to the original lateral connections of FPN, resulting in position attention guided connection (PAC) modules. Different to the existing spatial attention and channel attention mechanisms, the proposed PAC module generate a position weight matrix by mapping the feature map in the bottom-up path to the hidden space through a convolutional layer and then calculating the dependency between different regions on the converted feature maps; the position weight matrix is then multiplied with the feature maps generated by upsampling in the top-down pyramid to perform a positional recovery on the offset object. The novel position weight matrix is effective, because it is computed from the original features with accurate position information, and it can enhance the features in the region of the exact position of the object on the top-down feature map and suppress the features in other regions to perform the position retraction of the offset object.

The other advantage of PAC-Net is to propose a new Vertex Distance IoU (VDIoU) loss to solve the existing problem of inaccurate calculation of IoU-based losses. IoU loss is simply a way to determine the quality of RPs based on IoU, but it has the same loss for different RPs in many cases, thus affecting the learning ability of the network. The existing IoU-based losses are all based on adding new penalty terms to the IoU losses, where the common GIoU takes the non-overlapping area of the minimum enclosing box between RP and GT as the penalty term, and the other common DIoU takes the distance between RP and GT centroid as the penalty term. However, when RPs and GTs contain each other, GIoU degenerates to a common IoU. When RPs have the same centroid distance as GTs, DIoU cannot accurately evaluate the quality of these RPs, because they have the same DIoU loss. Based on DIoU, CIoU solves the problem of DIoU by introducing aspect ratio as an additional penalty term. However, since the calculation of CIoU involves inverse trigonometric functions, its computational complexity largely leads to its use outweighing the loss. Therefore, we propose the Vertex Distance IoU (VDIoU) loss: it builds on the IoU loss by calculating the sum of the distances between RP and the four vertices of GT and then dividing it by the diagonal distance of the minimum enclosing box as a penalty term. It not only takes into account the distance between RP and GT but also indirectly takes into account the aspect ratio by dividing the diagonal distance of the minimum enclosing box. Therefore, it does not have the problems of GIoU or DIoU, and its calculation is much simpler than CIoU. In this work, we fuse PAC-Net as the backbone of the Faster-RCNN detection framework to improve the performance of medical image detection. Actually, PAC-Net can also be applied as a feature learning backbone in other detection models, such as YOLO and RetinaNet, to help them perform better.

The main contributions of this paper are as follows:

• We identify the existing shortcomings of FPN-based detection models, the problem of object position offset and IoU-based losses calculation inaccuracy, and propose a novel Multi-Pathway FPN with Position Attention Guided Connections and Vertex Distance IoU model, PAC-Net, to achieve more accurate 3D medical image detection.
• We first propose a new position attention blocks into the lateral connections of FPN to generate a position weight matrix, thus importing positional recovery to resolve the object position offset problem in the upsampled feature map. Then, a vertex distance IoU (VDIoU) loss is further proposed by calculating the distance between the vertices of RP and GT divided by the diagonal length of the minimum enclosing box as the penalty term of IoU loss, which avoids the problems of inaccurate calculation (DIoU and GIoU) or large computational complexity (CIoU) in IoU-based loss calculation.
• We have conducted extensive experimental studies on the DeepLesion dataset (Ke et al., 2018), and the results show that: i) The PAC-Net significantly outperforms the state-of-the-art FPN-based detection baselines on the DeepLesion dataset. ii) The proposed improvement modules, PAC and VDIoU loss, are all effective and essential for PAC-Net to achieve a superior performance on the DeepLesion dataset. iii) The proposed VDIoU loss converges more quickly than the existing IoU-based losses, making PAC-Net an accurate and also highly efficient 3D medical image detection model.

## 2 Related work

### 2.1 Automatic medical image detection

Current deep-learning-based object detection models can be mainly divided into two categories: 1) two-stage models, e.g., Faster-RCNN (Ren et al., 2015), and 2) one-stage models, e.g., YOLO (Redmon et al., 2016). Faster R-CNN is developed based on the RCNN, which retains the overall framework of R-CNN (Girshick et al., 2014) and then uses the region proposal network (RPN) instead of selective search (SS) to generate region proposals for input to subsequent networks for classification and regression. By using RPN, Faster R-CNN achieves a faster and more accurate detection. Various extensions of Faster R-CNN have been proposed, such as Cascade R-CNN (Cai and Vasconcelos, 2018) and Mask R-CNN (He et al., 2017). Unlike the two-stage models, YOLO, the best-known one-stage method, uses a unified network to directly predict the entire feature map instead of the RPs for classification and bbox prediction, thus substantially improving the speed of detection. Based on this, a wide range of other one-stage models or frameworks have been proposed, such as YOLOv2 (Redmon and Farhadi, 2017), YOLOv3 (Redmon and Farhadi, 2018), and RetinaNet (Lin et al., 2017b).

All the above models have been very widely used for automated medical image detection. However, in order to utilize multi-scale features to better learn detailed information in medical images, most of these works use FPN instead of conventional CNNs for feature learning. For example, (Zlocha et al., 2019), used FPN as the backbone of RetinaNet and weak RECIST labels as auxiliary supervision to achieve the detection of lesions in CT images. PFA-ScanNet (Zhao et al., 2019) was proposed to use FPN to extract local and global features of different receptive fields to achieve automatic detection for cancer metastasis from whole slide images (WSIs). Besides, (Tsai and Peng, 2022), used FPN for network feature extraction, and then used a dual-head multi-task supervision approach with global and local labels to improve the feature learning capability of the network, and then took different data enhancement approaches for different task heads to make the different heads of the network achieve good results thus improving the lung nodule detection capability (Sheoran et al., 2022). Propose a robust single-stage FPN-based anchor-free lesion detection network, which can be improved by using the prediction of boxes that can be correlation-ranked according to their centers rather than overlap with objects to achieve good detection capability over different lesion sizes. In addition, to fully use the rich spatial context information in 3D medical data (Li et al., 2019), proposed an FPN-based MVP-Net using three-pathway input to help the network learn the detailed features better, which led to a good result on 3D CT lesion detection. Our work is similar to MVP-Net, but the significant difference is that MVP-Net assists the network in improving the detection accuracy by adding the *z*-axis position information of slices as additional information. At the same time, it does not address the position offset problem that exists in the FPN network structure. Although our work also focuses on automatic medical image detection, we identified the positional offset and IoU-based loss function degradation problems in existing works and propose a position attention guided connection (PAC) module and the vertex distance IoU (VDIoU) loss to solve these problems, respectively.

### 2.2 Attention mechanism

The attention mechanism is widely used in target detection tasks to help the network pay more attention to those important regions to improve detection results (Xu et al., 2022; Xu et al., 2019). (Zhou et al., 2019) uses the detection GT as a segmentation label and learns its features as an attention map to fuse to the original feature map to suppress the background to improve detection results (Huang et al., 2020). Transposes the gradient-based attention tensor over space and channels to generate inverted attention feature maps urging the network to detect objects based on parts of the object that are not too sensitive (Zhu et al., 2018). Combines attention-related information with global and local information about the object to improve detection performance by using a cascaded attention structure to perceive the global attention graph and encoding the attention graph into the network to obtain local perceptual features of the object (Lin et al., 2021). Propose an attention regularization module for the properties of local and global consistency and mutual knowledge transfer, using class activation maps (CAMs) of image-segmentation pairs to discover additional supervision in the regression network, and CAMs can act as augmentation gates for regions of interest, which in turn facilitate the segmentation task. Differently from these works, we focus on medical image detection tasks, and our position attention is targeted at the position offset problem encountered during upsampling operations in FPN. We compute the correlation between different regions based on the feature map without position offset to generate a position weight matrix to help recover the position offset problem.

### 2.3 IoU-based loss function

The IoU-based loss function is widely used in target detection tasks. The original IoU loss (Yu et al., 2016) is used as an evaluation criterion for the quality of region proposals by calculating the cross-merge ratio between RP and GT. Based on IoU loss, other methods of IoU-based loss add other judging factors as penalty terms to calculate the loss function. GIoU loss (Rezatofighi et al., 2019) takes the non-overlapping area of the minimum enclosing box between RP and GT as the penalty term. DIoU loss (Zheng et al., 2020) takes the distance of the centroid between RP and GT as the penalty term. On top of DIoU loss, CIoU loss (Zheng et al., 2020) additionally takes the similarity of the aspect ratio of RP and GT as the penalty term. However, GIoU and DIoU loss will degenerate to ordinary IoU loss in the face of some situations, and the high computational complexity of CIoU loss is not conducive to the training speed. Therefore, we propose VDIoU loss, which solves the above problem and has a low computational complexity by calculating the distance between RP and GT vertices divided by the diagonal length of the minimum enclosing box as the penalty term.

## 3 Methods

**FIGURE 1:**
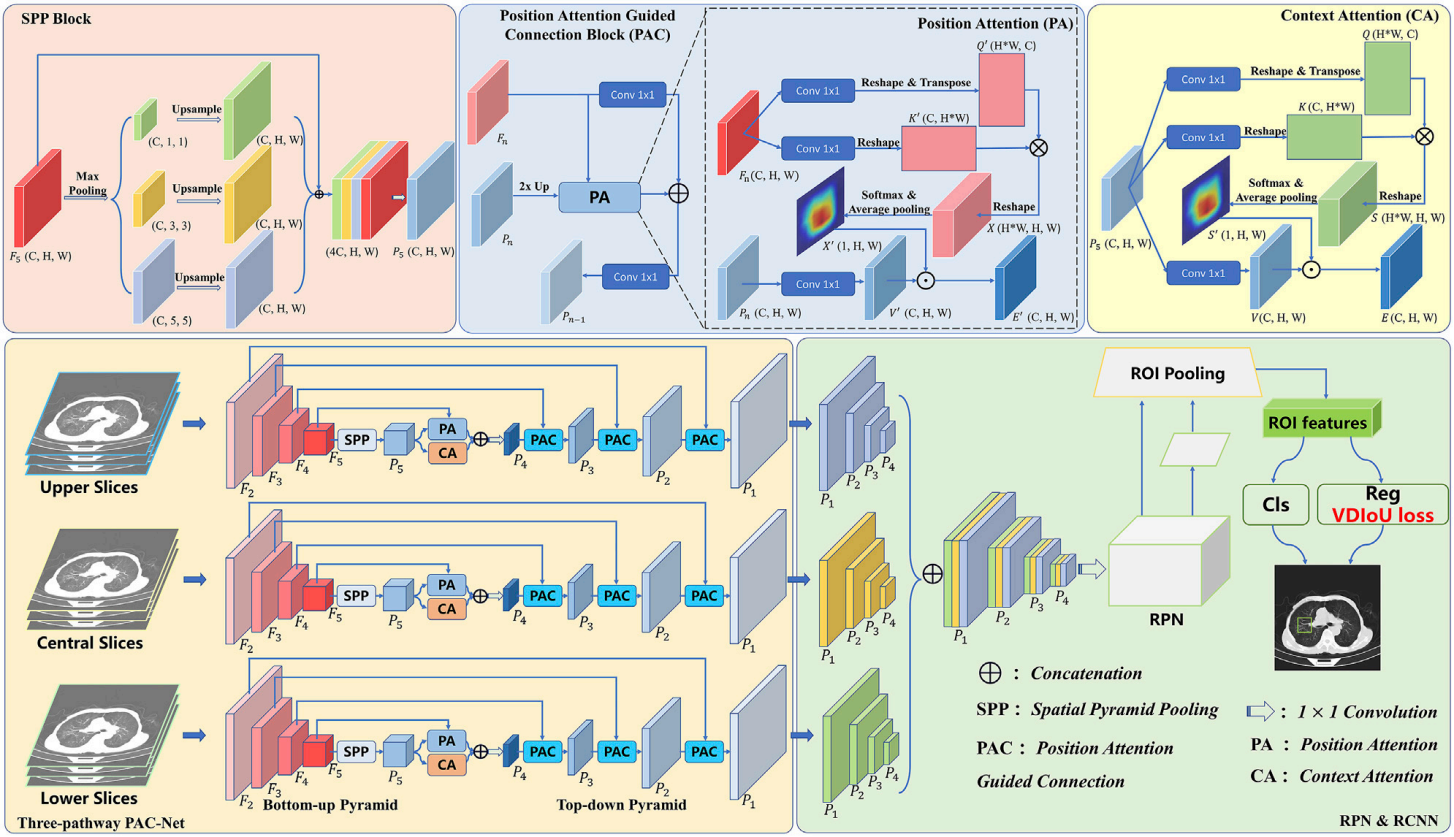
Overview of incorporating PAC-Net into Faster R-CNN for 3D medical image detection.

In order to achieve accurate medical image detection, in this work, a novel FPN-based model, called Multi-Pathway FPN with Position Attention Guided Connections and Vertex Distance IoU Loss (PAC-Net), is proposed. As shown in Figure 1, we use the proposed PAC-Net instead of FPN as the backbone of the Faster R-CNN to extract features and then feed the features into the subsequent RPN and R-CNN networks and use the VDIoU loss to calculate the regression loss for training. Compared to the conventional FPN, PAC-Net mainly consists of two additional advanced modules: SPP block and position attention guided connection (PAC) blocks. For better feature extraction, we add the SPP block to the last layer of FPN’s bottom-up, since it significantly increases the receptive field and separates out the most significant context features. At the same time, in order to solve the position information offset problem caused by the up-sampling operation in the lateral connection in FPN and the SPP block, we add position attention (PA) to each lateral connection of the FPN and the SPP block to form a position attention guided connection (PAC) block. Specially, in order to better focus on important regions, we also add a self-attention-based context attention (CA) module to the top-level features after SPP to enable them to focus more on important regions through the correlation weights of global features. PA recovers the position of the offset object by multiplying the position weight matrix calculated from the original features with accurate position information with the feature map generated by upsampling in a top-down pyramid. Specifically, features with high-level semantic information and features with accurate location information are combined to obtain a more comprehensive representation by refining the PAC structure composed of CA and PA jointly at the top layer of the FPN. Furthermore, the PAC composed of PA alone is used in the lateral connection of FPN to solve the problem of position information offset caused by the upsampling operation. Besides, we also use VDIoU loss instead of IoU-based loss to solve the possible inaccurate evaluation problem.

In general, we use the slices corresponding to 3D medical images with annotations as the central slices and the adjacent slices as the upper and lower slices. The three sets of slices are input to the three-pathway PAC-Net, and then the features with larger receptive fields are obtained with the SPP block after multiple convolutions by the bottom-up pyramid. These features are fed into a top-down pyramid for upsampling, and then passed through a PAC composed of CA and PA multidimensional attention at the top level, and fused with the corresponding feature maps with precise spatial location information through a PAC composed of separate PA after each upsampling to better learn the important information including semantic and location information. Then, the features of the corresponding prediction layers of the three pathways are concatenated together and fed into a 1 × 1 convolution layer. Finally, the features are fed into the RPN and R-CNN networks for lesion detection by calculating the VDIoU regression loss and classification loss. Details of these advanced modules will be presented in the rest of this section.

### 3.1 Position attention guided connection module

Although the features from the SPP block contain rich receptive field information, not all of them are useful to facilitate the performance of object detection. The accuracy may be reduced due to bounding box or area suggestions being misleading by redundant information, and also due to the offset of object position information caused by upsampling operations. Thus, to remove the negative impacts of the redundancy and further enhance the representation ability of feature maps, we propose a Position Attention Guided Connection (PAC) Module, which can capture salient dependencies with strong semantics and precise locations. As shown in Figure 1, in the top layer of the FPN, the PAC module consists of two parts: i) the positional attention module (PA) and ii) the context attention module (CA); and in the lateral connection of the FPN, the PAC module consists of PA alone.

Specifically, in the top-level PAC module, CA focuses on the semantics between subregions of a given feature map (i.e., features from the SPP layer). However, the position information of each object is offset due to the upsampling operation. To alleviate this problem, we introduce PA, which focuses more on guaranteeing spatial information. Finally, the features refined by CA and PA are combined to obtain a more comprehensive representation. Furthermore, in the laterally connected PAC module, features with high-level semantic information are combined with the accurate position information of the shallow features extracted by the PA module to ensure that the features can obtain a more comprehensive representation.

#### 3.1.1 Context attention

To actively capture the semantic dependencies between subregions, we introduce a context attention (CA) module based on the self-attention mechanism. We feed the preceding features, which contain multi-scale receptive field information, into the CA module. Based on these informative features, CA adaptively pays more attention to the relations between subregions, which are more relevant. Thus, the output features from CA will have clear semantics and contain contextual dependencies within surrounding objects.

As can be seen in Figure 1, given discriminative feature maps $P_{5}\in R^{C\times H\times W}$ , we transform them into a latent space by using the convolutional layers **W**_*q*_ and **W**_*k*_, respectively. The converted feature maps are calculated by

$$Q=W_{q}^{⊤}P_{5}\;\;\text{and}\;\;K=W_{k}^{⊤}P_{5},$$ (1)

where $\left\{Q,K\right\}\in R^{C\times H\times W}$ . Then, we reshape **Q** and **K** to $R^{C\times HW}$ . To capture the relationship between each subregion, we calculate a correlation matrix as

$$S=Q^{⊤}K,$$ (2)

where $S\in R^{HW\times HW}$ and then reshape it to $R\in R^{HW\times H\times W}$ . After normalizing **S** *via* softmax activation function and average pooling, we build an attention matrix **S**′, where $S^{\prime }\in R^{1\times H\times W}$ . Meanwhile, we transform the feature map **F** to another representation **V** by using the convolutional layer **W**_*v*_:

$$V=W_{v}^{⊤}P_{5},$$ (3)

where $V\in R^{C\times H\times W}$ . Finally, an element-wise multiplication is performed on **S**′ and the feature **V** to get the attentional representation **E**. We formulate the function as

$$E=S^{\prime }⊙V.$$ (4)

By calculating the correlation between the subregions in the feature map containing multi-scale information, the network is able to pay more attention to the contextual information and thus focus more on those key regions to improve the detection results.

#### 3.1.2 Position attention

Due to the effects of upsampling, the position information in the feature map is offset, thus affecting the detection accuracy. To solve this problem, we propose a new attention module, called position attention (PA) module, which uses the accurate position information in the unsampled feature maps to guide deep features with rich contextual information to obtain feature maps that maintain high-level semantic information while having accurate position information.

As shown in Figure 1, similarly to CA, we use convolutional layers to transform the given feature maps. Different from the CA, the input of the PA consists of two parts, which are **P**_*n*_ rich in high-level semantic information and the corresponding feature map **F**_*n*_ with accurate position information for calculating the weight matrix. To get the attention matrix, first, we apply two convolutional layers $W_{q}^{\prime }$ and $W_{k}^{\prime }$ , to convert **F**_*n*_ into the latent space, respectively:

$$Q^{\prime }=W_{q^{\prime }}^{⊤}F_{n}\;\;\text{and}\;\;K^{\prime }=W_{k^{\prime }}^{⊤}F_{n},$$ (5)

where $\left\{Q^{\prime },K^{\prime }\right\}\in R^{C\times H\times W}$ . Then, we reshape the dimension of **Q**′ and **K**′ to $R^{C\times HW}$ , and produce the correlation matrix similar to Eq. 2 as:

$$X={Q^{\prime }}^{⊤}K^{\prime },$$ (6)

where $X\in R^{HW\times HW}$ . After reshaping **S** to $R^{HW\times H\times W}$ , we employ softmax function and average pooling to produce an attention matrix $X^{\prime }\in R^{1\times H\times W}$ . To obtain a prominent representation, we combine the extracted features **V**′ with **X**′ by element-wise multiplication:

$$E^{\prime }=X^{\prime }⊙V^{\prime }.$$ (7)

The weight matrix extracted by shallow features that have accurate location information can enhance the features in the region of the exact position of the object on the feature map and suppress the features in other regions to perform the position retraction of the offset object. Therefore, the feature map with rich high-level semantic information but offset location information can be repaired by PA to obtain a feature map with more comprehensive representation capability.

#### 3.1.3 Position attention guided connections

As mentioned earlier, the FPN fuses the high-resolution feature maps containing location information with the low-resolution feature maps containing more semantic information by lateral concatenation to preserve the location information in the feature maps as much as possible. However, the position information in the later feature maps with rich semantic information has been changed due to a large number of upsampling operations, which affects the detection accuracy. Therefore, we add a PA module in the lateral connection, aiming to let the high-resolution feature map with a lot of position information guide the low-resolution feature map with more semantic information to recover the corrupted position information as much as possible. Specifically, in the lateral concatenation of each layer, we use the feature map **P**_*n*_ rich in high-level semantic information but with a offset in the position information and the corresponding feature map **F**_*n*_ containing accurate position information as the input of the PA. Then, we concatenate the obtained result with the feature map **P**_*n*_ after 1 × 1 convolution. Finally, after 1 × 1 convolution, the output feature map **P**_**n**−**1**_ is obtained as the prediction feature map of this layer and the input of the next layer.

### 3.2 VDIoU loss

Based on the original IoU, many evaluation factors are derived from enriching the evaluation dimensions of previous IoU from those different aspects. The original IoU loss only considers the calculation of overlapping areas. Its calculation formula is as follows:

$$L_{\mathrm{IoU}}=1-\frac{GT\cap RP}{GT\cup RP}.$$ (8)

**FIGURE 2:**
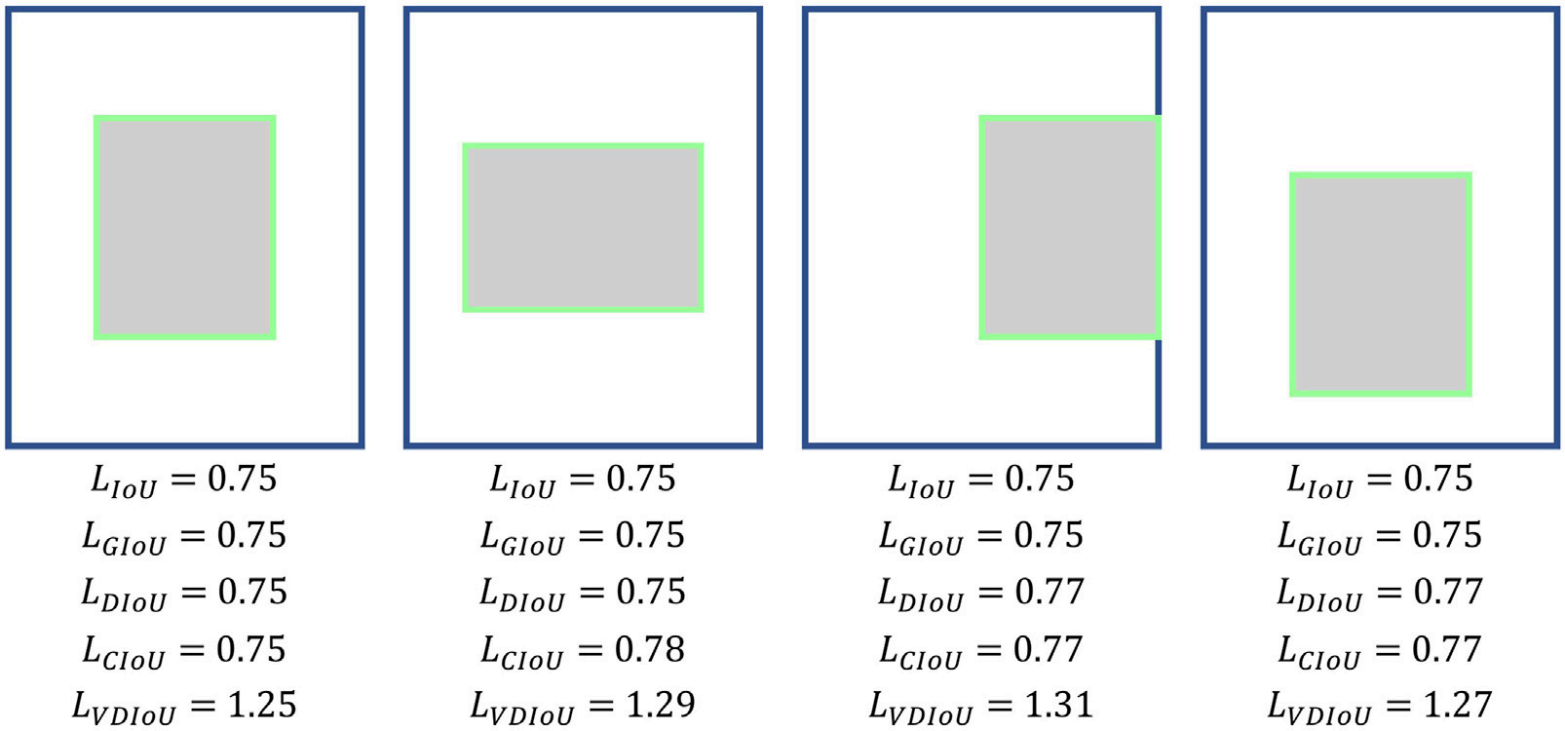
Calculation of the existing IoU-based losses.

**FIGURE 3:**
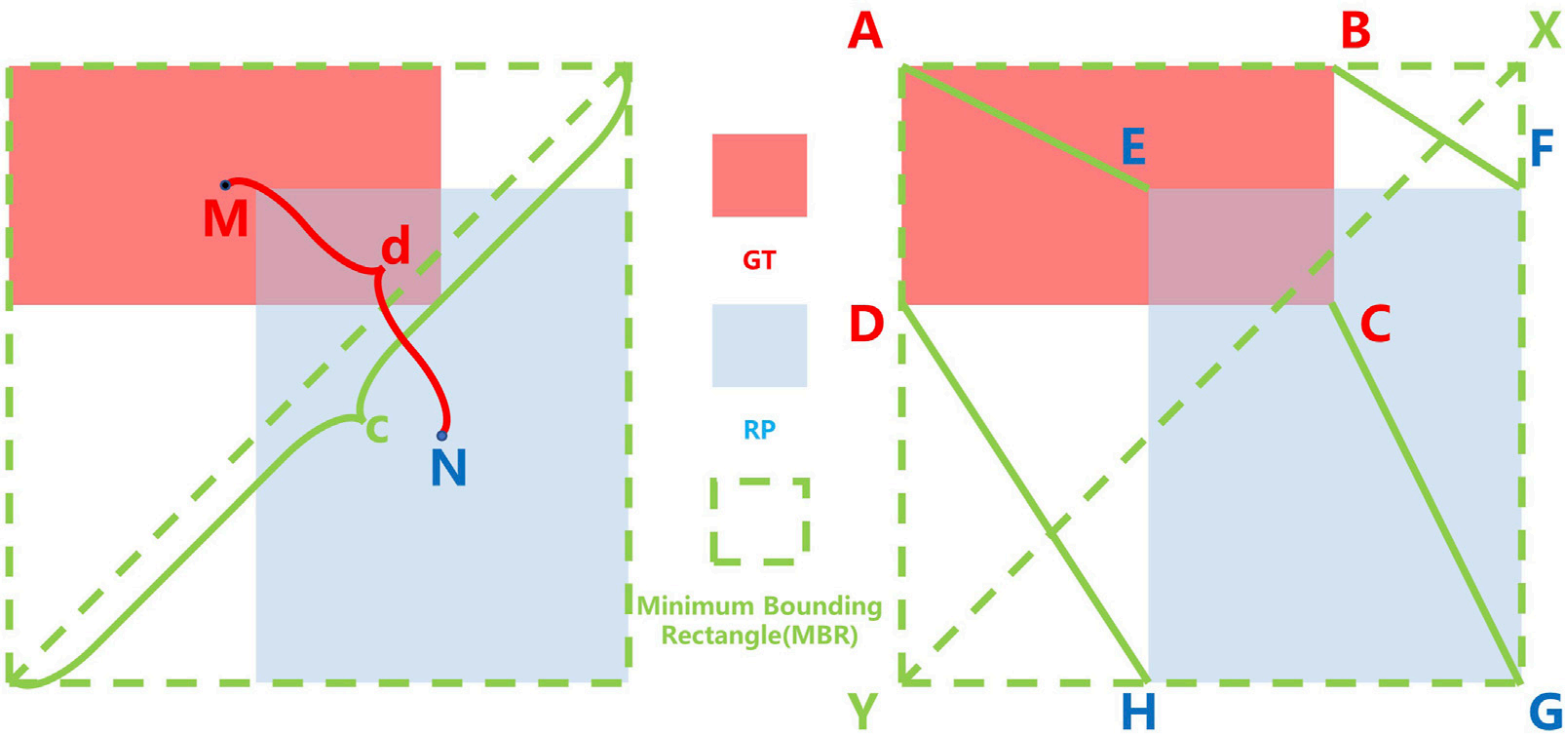
Calculation of IoU-based loss and Vertex Distance IoU (VDIoU) loss, where the red, blue, and green dotted boxes represent the Ground Truth (GT), Region Proposal (RP), and Minimum Bounding Rectangle (MBR), respectively; M, N is the central point of GT and RP, respectively; c is the distance between GT and the RP center point and d is the diagonal length of the MBR; A, B, C, D and E, F, G, H are the vertices of GT and RP, respectively.

Only the overlapping area does not accurately judge the quality of a region proposal (RP). Therefore, IoU loss has some errors in the evaluation of a RP in some cases. Meanwhile, GIoU pays attention to the overlapping and non-overlapping areas, strengthening the discussion of the evaluation system. Its calculation formula is as follows:

$$L_{\mathrm{GIoU}}=L_{\mathrm{IoU}}+\frac{MBR-GT\cup RP}{MBR},$$ (9)

where MBR is the area of the Minimum Bounding Rectangle as shown in Figure 3. However, GIoU obviously ignored the measurement of the difference between RP and GT. The measurement of the difference between RP and GT include the distance between the center points and the ratio of length-width. Ignoring these factors will result in GIoU not being able to assess how good an RP is either truly. As shown in Figure 2, when the RPs are in different locations in the GT interior, they have the same IoU and GIoU loss, but obviously, the quality of these RPs is different. A good loss for bounding box regression should consider three important geometric measures, i.e., overlap area, central point distance, and aspect ratio, which have been ignored for a long time. By combining these geometric measures, The Distance-IoU (DIoU) loss was proposed for bounding box regression, leading to faster convergence and better performance than IoU and GIoU losses. Its calculation formula is as follows:

$$L_{\mathrm{DIoU}}=L_{\mathrm{IoU}}+\frac{d^{2}}{c^{2}},$$ (10)

where d is distance between the center points of GT and RP and c is the diagonal length of the Minimum Bounding Rectangle (MBR) of RP and GT as shown in Figure 3. But DIoU loss cannot distinguish which region proposals are more similar to ground truth when the center points of the region proposals are at the same position. Furthermore, based on DIoU, CIoU uses the similarity of the aspect ratio of RP and GT as the evaluation factor, which enables CIoU to be more accurate in evaluating the quality of a RP. Its calculation formula is as follows:

$$L_{\mathrm{CIoU}}=L_{\mathrm{IoU}}+\frac{d^{2}}{c^{2}}+\alpha \upsilon ,$$ (11)

where *υ* is:

$$\upsilon =\frac{4}{\pi^{2}}{\left(\arctan\left(\frac{w^{GT}}{h^{GT}}\right)-\arctan\left(\frac{w^{RP}}{h^{RP}}\right)\right)}^{2},$$ (12)

where, as in Figure 3, *w* ^*GT*^, *h* ^*GT*^ are the width and height of GT, and *w* ^*RP*^, *h* ^*RP*^ are the width and height of RP. And *α* is:

$$\alpha =\frac{\upsilon }{L_{\mathrm{IoU}}+\upsilon }.$$ (13)

However, its high computational complexity due to the use of the inverse trigonometric function calculation will slow down the training speed. Moreover, it can also fail in some cases. As shown in Figure 2, when the aspect ratio between RP and GT is fixed and the centroid distance is the same, the different positions of RP in GT do not affect the results of several IoU losses mentioned above, but it is obvious that these boxes have different qualities.

Therefore, based on the original IoU loss functions, we propose Vertex Distance IoU (VDIoU) loss functions in this paper. The VDIoU loss function is obtained by taking the ratio of the sum of the distances between the four vertices corresponding to RP and GT and the diagonal length of the minimum enclosing frame as an additional penalty term of the IoU loss. The VDIoU loss function converges faster and greatly reduces the complexity of the operation. Specifically, Vertex Distance Intersection over Union (VDIoU) loss is an evaluation method that indirectly examines the similarity of RP and GT by not directly measuring the similarity of distance and shape between their centroids but by using these two rubrics in an indirect way. The VDIoU-loss-specific formula is shown below:

$$L_{\mathrm{V DIoU}}=L_{\mathrm{IoU}}+VDIoU,$$ (14)

where $L_{\mathrm{IoU}}$ is the original IoU loss, and the additional penalty term VDIoU is calculated as shown in Figure 3. AE, BF, CG, and DH are the distances between the corresponding four vertices of RP and GT, and XY is the diagonal length of the Minimum Bounding Rectangle (MBR) of RP and GT. The sum of the distances between the four vertices is divided by two times the diagonal length of the MBR to obtain VDIoU, as shown in the following equation:

$$VDIoU=\frac{AE+BF+CG+DH}{2XY}.$$ (15)

By observing this equation, we can intuitively feel that in the process of backpropagation, the model tends to pull the four vertices of the region proposal toward the four vertices of the ground truth until they overlap. In this process, the difference in centroid distance and aspect ratio between RP and GT decreases simultaneously. Although the formula of VDIoU does not mention centroid distance and aspect ratio, its calculation result is directly affected by the centroid distance and aspect ratio. So the final calculation result reflects a measure of the degree of difference between RP and GT. A lower value of VDIoU represents a higher degree of similarity between RP and GT.

### 3.3 SPP block

Generally, with a deeper network, the receptive field will be larger, and it is easier to extract the global feature information. However, the performance of object detection is not simply positively correlated with the receptive field. The deep features contain more object category information, and the shallow features contain more object location information. Therefore, fusing the features of different receptive fields and the features of sub-regions can enhance the feature representation ability of the network (Wang et al., 2019). Therefore, we added the SPP block to the network to effectively use multi-scale feature information to enhance the feature representation ability of the overall network.

## 4 Experiments

### 4.1 Datasets

In order to evaluate the performance of our proposed PAC-Net for lesion detection tasks on 3D medical images, we conduct extensive experiments on the DeepLesion dataset^1^, the largest publicly available dataset of multi-class, lesion-level annotated clinical medical CT images. It is a large-scale dataset with 32,735 lesions distributed over 32,120 axial slices from 10,594^−ΔΔCT^ studies of 4,427 unique patients. The dataset provides not only the key CT slice containing the lesion but also its 3D context (additional slices of 30 mm above and below the key slice, for a total of 928,020^−ΔΔCT^ images). Furthermore, the dataset provider has already divided the dataset into three subsets, i.e., 15% of the dataset is used as the validation set (4889 lesions), another 15% is used as the test set (4927 lesions), and the rest is used as the training set (22919 lesions). In addition, unlike existing datasets that typically focus on one type of lesions, DeepLesion includes several different types of lesions, e.g., lung, liver, kidney, etc.; therefore, to better evaluate the performances of different methods under the detection tasks of different types of lesions, the dataset provider also further divides the validation set and test set, according to the different lesion detection tasks at different body parts, into eight subsets, i.e., lung (LU), abdomen (AB), mediastinum (ME), liver (LV), pelvis (PV), soft tissue (ST), kidney (KD), and bone (BN), respectively. Almost all the existing works directly adopt these two official divisions of the dataset in their experiments; to keep fair comparison, these two official divisions are also adopted for all the methods (including the proposed PAC-Net and all the baselines) in our work. The number of validation sets and test sets contained in different subsets is shown in Table 1. We uniformly resize the images to 512*512.

**TABLE 1:** Dataset information.

	LU	ME	LV	ST	PV	AB	KD	BN	Easy	Medium	Hard
Validation set	1294	808	584	458	342	1003	261	139	2686	1345	858
Test set	1100	864	700	409	339	1173	234	108	2664	1512	751
Total	2394	1672	1284	867	681	2176	495	247	5350	2857	1609

To better show the performance of different methods under different detection difficulty tasks, we further combine these eight subset parts into three types of different difficulty detection subtasks, i.e., easy, medium, and hard detection tasks. The easy detection task represents the task with a high detection accuracy of existing methods, which combines three easy detection subsets, LU, ME, and LV; the medium detection task represents the task with an average detection accuracy of existing methods, which combines two medium detection subsets, PA and AB; the hard detection task represents the task with a poor detection accuracy of existing methods, which combines three subsets with high detection difficulty, BN, KD, and ST. The number of validation sets and testing sets contained in the three types of different difficulty detection subtasks is also shown in Table 1. Please note that all the experimental results of all methods shown in the same table or figure in our work are obtained by experiments under the same dataset division.

### 4.2 Baseline

Four state-of-the-art deep models on natural image detection, i.e., Faster R-CNN, Cascade R-CNN, YOLOv3, and RetinaNet, are used as the baselines. All the above models use FPN with ResNet-50 as the backbone. Besides, we also compare our proposed PAC-Net with two state-of-the-art methods on the DeepLesion dataset, i.e., 3DCE (Yan et al., 2018) and MVP-Net. MVP-Net uses FPN to improve detection accuracy using multi-scale information, while 3DCE uses R-FCN. For a fair comparison, we replace R-FCN with FPN in 3DCE. As before, these methods use ResNet50 as the backbone.

### 4.3 Evaluation metrics

The free-response receiver operating characteristic (FROC) curves allow the evaluation of arbitrary abnormalities on each image and are therefore often used in medical detection tasks. Specifically, the detection of medical images requires an extremely high recall rate and therefore tolerates a certain number of false positives on a single image. Therefore, for most medical detection tasks, sensitivity at different false positives (FPs) on each image is a common evaluation metric. Furthermore, in order to show the superior performances of our proposed model more comprehensively, we show the sensitivity results at 0.125, 0.25, 0.5, 1, 2, 3, 4 and 8 FPs per image on the DeepLesion dataset in Table 2. However, although the sensitivity will increase with higher FPs, setting too high FPs will result in too many false positive boxes in the image, which thus greatly interfere the diagnosis and is inapplicable in the clinical practices. Therefore, most of the existing works use the sensitivity at 2 and 4 FPs as evaluation metrics, e.g., (Tang et al., 2019), (Yan et al., 2018), and (Li et al., 2019). Therefore, in this work, to keep fair comparison, we follow this setup and report the sensitivity at 2 and 4 FPs in Tables 3–Tables 5. In addition, we use mAP, which is commonly used on natural images, as an evaluation metric. The mAP is calculated by computing the average of APs with an IoU threshold of 0.5–0.95. Meanwhile, the *p*-value is used to measure the significance of improvements.

**TABLE 2:** Comparison with state-of-the-art object detection on DeepLesion. Sensitivity at various FPs per image and mAP on the test set of the official data split of DeepLesion. IoU criteria and 3 slices input (one key slice and two neighbor slices) are used.

	Sensitivity IoU (%)								Mean of	mAP
	0.125	0.25	0.5	1	2	3	4	8	[0.125–8]	(%)
Faster R-CNN	25.65	36.25	48.60	60.57	71.19	74.53	76.51	84.77	59.76	20.8
Cascade R-CNN	25.29	36.55	48.93	61.24	72.15	74.66	76.72	84.81	60.04	21.1
YOLO-v3	24.52	35.70	46.19	59.60	69.65	72.37	75.21	82.18	58.17	19.6
RetinaNet	24.89	36.31	47.26	59.90	70.68	72.57	75.34	83.15	58.76	20.1
3DCE, 3 slices	32.55	44.03	56.49	67.85	76.89	80.56	82.76	87.03	66.02	25.6
FPN+3DCE, 3 slices	33.82	46.84	58.85	69.44	78.90	81.13	83.82	87.26	67.51	28.2
MVP-Net, 3 slices	44.20	57.46	69.39	77.91	84.05	88.13	88.75	89.86	74.97	34.6
Ours, 3 slices	49.25	62.90	73.19	81.12	86.91	88.76	91.37	92.21	78.21	36.3

**TABLE 3:** Sensitivity (%) at 2 and 4 false positives per image on the eight subsets of DeepLesion.

	LU		ME		LV		ST		PV		AB		KD		BN	
	2	4	2	4	2	4	2	4	2	4	2	4	2	4	2	4
Faster R-CNN	71.83	76.62	72.06	77.13	71.80	77.96	66.67	70.95	71.12	75.88	71.43	76.25	54.83	60.62	46.79	51.72
Cascade R-CNN	72.03	76.77	72.18	78.01	71.97	78.15	67.12	71.24	71.35	75.99	72.11	76.85	55.21	61.37	48.12	52.19
YOLO-v3	70.25	75.69	70.12	76.16	69.83	75.42	65.57	70.18	68.96	74.75	69.36	74.87	54.37	59.30	46.03	50.72
RetinaNet	70.85	76.13	71.56	76.65	70.24	76.39	66.49	70.59	69.52	75.48	70.55	75.37	54.26	59.64	46.77	51.15
3DCE, 3slices	76.03	82.43	77.13	83.34	76.05	83.09	70.18	75.69	76.67	81.38	77.93	83.01	59.57	65.09	50.64	57.90
FPN+3DCE, 3slices	79.29	83.82	78.89	84.12	77.36	83.65	71.38	76.49	78.30	83.71	79.47	84.80	60.64	68.46	50.78	58.43
MVP-Net, 3slices	84.72	89.10	84.62	86.41	83.94	88.99	77.24	79.67	83.07	86.64	85.41	87.14	65.51	69.70	51.52	57.38
Ours, 3slices	87.13	91.66	87.68	91.77	88.59	91.53	77.89	81.38	85.12	87.52	86.53	91.59	65.09	68.46	63.36	67.33

**TABLE 4:** Sensitivity (%) at 2 and 4 false positives per image on DeepLesion’s three subsets of detection difficulty.

	Easy (FPs)		Medium (FPs)		Hard (FPs)	
	2	4	2	4	2	4
Faster R-CNN	71.90	77.13	71.36	76.17	60.12	64.97
Cascade R-CNN	72.06	77.53	71.94	76.66	60.68	65.43
YOLO-v3	70.10	75.78	69.27	74.84	59.27	63.99
RetinaNet	70.92	76.37	70.32	75.39	59.84	64.38
3DCE, 3 slices	76.39	82.90	77.65	82.64	64.06	69.83
FPN+3DCE, 3 slices	78.65	83.87	79.21	84.56	65.07	71.39
MVP-Net, 3 slices	84.48	88.20	84.89	87.02	69.89	73.36
Ours, 3 slices	87.69	91.67	86.21	90.68	71.81	75.33

**TABLE 5:** Ablation study of our approach on the DeepLesion dataset.

FPN	SPP layer	PAC	VDIoU loss	FPs@2.0	FPs@4.0
*✓*				78.90	83.82
*✓*	*✓*			80.57	85.16
*✓*	*✓*	*✓*		85.24	89.36
*✓*	*✓*		*✓*	82.68	86.53
*✓*	*✓*	*✓*	*✓*	86.91	91.37

### 4.4 Implementation details

Our experiments are implemented using the PyTorch framework and run on an NVIDIA GeForce RTX 2080Ti GPU. We use FPN with ResNet-50 for all experiments. The ResNet-50 backbone is initialized with an ImageNet pre-trained model, and all other layers are randomly initialized. Each mini-batch had 2 samples, where each sample consisted of three 3-channel images for 3D fusion. Anchor scales in RPN are set to (16, 32, 64, 128, 256) and anchor aspect ratios are set to (0.5, 1, 2). Differently from the upsampling in the original FPN, we use bilinear interpolation instead of nearest-neighbor interpolation. We used SGD to train PAC-Net and set the base learning rate to 0.004, then reduced it by a factor of 10 after 4 epochs. We train the network for 15 epochs with a batchsize of 2 to ensure that the network converges completely. Then the model with the best results on the validation set is selected for testing.

## 5 Results

In this section, we conduct extensive experiments to investigate the effectiveness of the proposed method in medical image detection tasks. We first compare our proposed PAC-Net with several state-of-the-art baseline methods on the DeepLesion dataset. After that, we further validate the effectiveness of important components of our model, including PAC, VDIoU loss, and SPP block. Extensive experimental results demonstrate the effectiveness of our proposed method and verify that each part of our proposed approach is efficient and significant.

### 5.1 Main results

The results on the entire DeepLesion dataset are shown in Table.2, while the results on the eight subclasses are shown in Table.3. Also, as mentioned earlier, to further validate the performance of our method under different detection difficulties, the results on three subclasses according to the difficulty are shown in Table.4. To investigate the effectiveness of our proposed PAC-Net, we compared the performance of PAC-Net with the baseline of four SOTA methods for natural image detection (Faster R-CNN, Cascade R-CNN, Yolo-v3, and RetinaNet) and two SOTA methods on DeepLesion (3DCE and MVP-Net). In addition, since the original implementation of 3DCE is based on R-FCN, we have re-implemented 3DCE as a baseline using the FPN backbone for a fair comparison.

In general, as shown in Table.2, we find that our proposed PAC-Net outperforms all baselines in general, which proves that our proposed PAC-Net is more accurate than the SOTA methods for 3D medical image detection. Specifically, we first find that the results of 3DCE improve a lot after using FPN instead of R-FCN as the backbone. This well demonstrates that FPN enables the network to learn features of different depths at different scales through multi-scale feature fusion to improve the final detection results. Then, we find that the results of the 3-slices input-based methods are much better than those of the single-slice input, because the 3-slices input can make full use of the spatial information of 3D medical images to learn richer features from them to help the network improve the feature representation. Finally, as shown in Table 2, the proposed PAC-Net achieves better detection performance than the SOTA methods 3DCE and MVP-Net in all evaluation metrics. We also calculate the *p*-value of the proposed model PAC-Net w.r.t. the state-of-the-art baselines, i.e., MVP-Net and 3DCE. Specifically, the *p*-value of our work w.r.t. the SOTA 3DCE model is 0.0422, which proves that our work achieves very significant improvements compared to 3DCE. Furthermore, the *p*-value of our work w.r.t. MVP is 0.2855; although it is higher than 0.05, it is also a remarkable improvement because it is not easy to always achieve statistically significant improvements in the deep learning related research areas, e.g., the *p*-value of MVP-Net compared to 3DCE is 0.1745 (also large than 0.05), and our work is further improving on the basis of the SOTA MVP-Net, which is more challenging. The reasons for our improvement compared to the previous method are the following: i) PAC-Net utilizes the position attention guided connection module to compensate for the object position offset problem caused by the upsampling operation on the FPN, ii) PAC-Net additionally utilizes the SPP block to effectively use multi-scale feature information to enhance the feature representation ability of the overall network, and iii) the improved VDIoU loss can also help the network training to be more accurate.

The DeepLesion dataset can be roughly divided into 8 different types of lesions. In Table.3, we show in detail the detection results of eight types of lesions. It can be seen that in these eight types of lesions, our detection accuracy has improved to varying degrees, and the accuracy of the most difficult to detect bones is the most obvious. This shows the effectiveness of our method, which proves that our network has fully learned the feature differences between different lesions and can cope with the difficult task of detecting different lesions.

In order to demonstrate more fully that our proposed method is able to achieve a degree of improvement even in the more difficult types of detection, we have divided these eight subsets of lesions into three subsets, the Easy set, the Medium set, and the Hard set, according to the difficulty of detection. For medical reasons, such as the low contrast between bone and surrounding tissues and the small target and variable morphology of soft tissues, the three types of lesions, BN, KD, and ST, are more difficult to detect overall, so we classify them in the Hard set. On the other hand, the AB, LU, and LV are easier to detect because of their greater contrast with surrounding tissue and larger detection targets, so we have placed these three subsets in the Easy set. The others are classified as the Medium set.

As before, we not only compared the results of the traditional methods on these three sets but also the SOTA methods’ results on the DeepLesion dataset. The results are shown in Table.4. It can be clearly seen that our results are the best no matter which method is compared. Furthermore, on the Hard set, which is the hardest to detect, we have the biggest improvement. This is because we can use the characteristics of different receptive fields by using multi-scale features, detecting larger lesions and learning from small ones. Moreover, 3D contextual information and an attention mechanism that retains position information can help the bboxes return more accurately. This effectively proves the effectiveness of our method.

### 5.2 Ablation study

To investigate the effect of the two proposed advanced modules, the position attention guided connection (PAC) module and the Vertex Distance IoU loss (VDIoU), and the additional added multi-scale module SPP layer in an ablation study, Table five shows the detection accuracy of the model on the DeepLesion dataset. Firstly, it can be seen that the detection results are improved after adding the multi-scale module SPP layer, which indicates that the additional multi-scale information fusion is still helpful for FPN.

#### 5.2.1 Effectiveness of PAC

**FIGURE 4:**
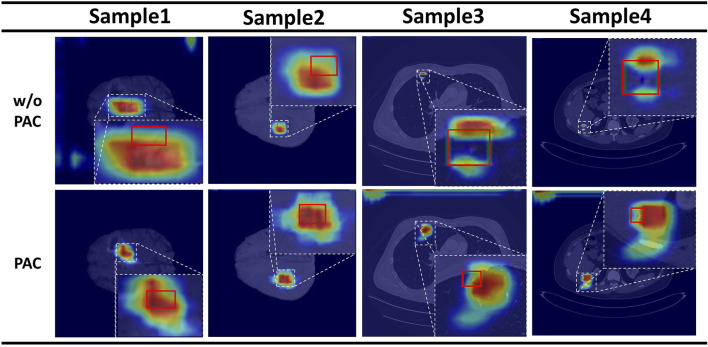
Results of the attention heat map. Above is the result without PAC, and below is the result with PAC.

We then compared the effectiveness of the PAC module on detection. The results show that FPN with PAC always outperforms vanilla FPN in the metrics with the help of PAC. Thus, this demonstrates that PAC can enhance features in the exact location region of the object and suppress features in other regions on the top-down feature map by means of a position weight matrix calculated from the original features with accurate position information to achieve position retraction of the offset object. Also, to verify the validity of our proposed attention, we not only did the above ablation experiment, but we also visualized the heat map of attention. As shown in Figure 4, the top is the visualization of the heat map without attention, while the bottom is the visualization of the heat map with attention added. The comparison between the two is still obvious, as we can clearly see that with the addition of attention, the model is able to focus more on the focal area and not on a scattered area.

#### 5.2.2 Effectiveness of VDIoU loss

Then, we compare the performance of FPN with VDIoU in Table 5. The results show that using VDIoU loss can improve the prediction accuracy of the model. This greatly supports our argument that using VDIoU loss to replace the IoU-based loss in the general detection model can solve the existing problem of IoU series loss and thus improve the accuracy.

#### 5.2.3 Effectiveness of using both PAC and VDIoU loss

Finally, we find that by using both PAC and VDIoU loss in Table 5, the results are better than using only one of them. This is because PAC and VDIoU loss are designed to solve different problems in FPN-based detection networks and can complement each other to improve the detection accuracy of the depth model. Consequently, all the above observations demonstrate that PAC-Net is an effective and efficient FPN-based backbone model, and both PAC and VDIoU loss are effective and essential for PAC-Net to achieve a superior performance.

### 5.3 Visualization results

**FIGURE 5:**
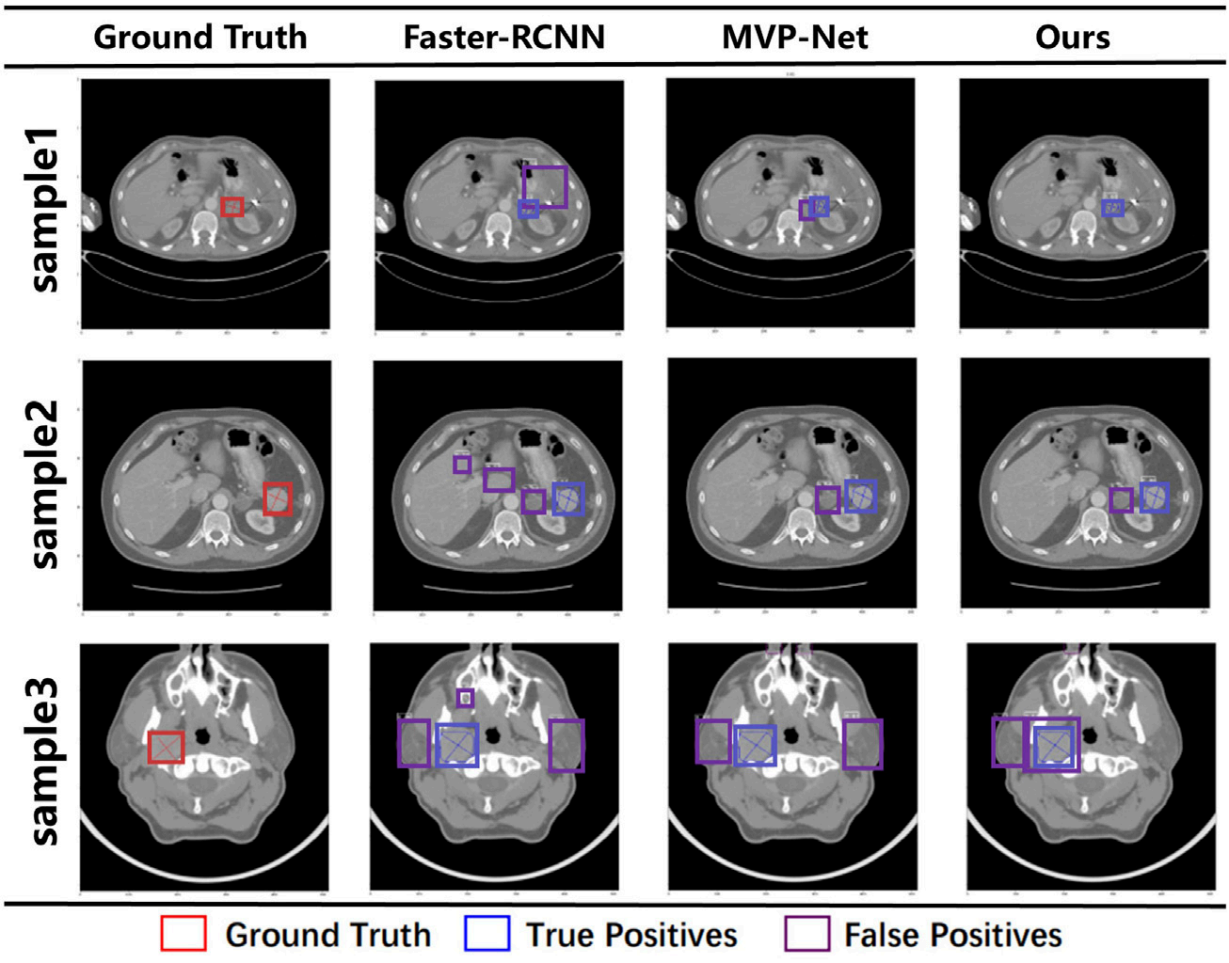
Visualization results under different models. The red box represents the ground truth, the blue box represents true positive, and the purple box represents false positive.

To compare the final detection results more visually, Figure 5 visualizes the final detection results of the different methods and our method, where GT is on the left and the last three columns are Faster R-CNN, MVP-Net, and our method. The red boxes on the graph represent Ground Truth (GT), the blue boxes represent True Positive (TP), and the purple boxes represent False Positive (FP). It is intuitive from the first line that all the other methods give redundant detection boxes, whereas ours does not. Furthermore, in the second three rows, although all the detections have redundant boxes, the positions of the boxes detected by our method are all around the GT, whereas the other methods give detection boxes in places that are not relevant. This indicates that our model can focus more accurately on the location of the lesion, so the detection boxes are more accurately located.

From the results, we can see that our results are the best compared with traditional target detection networks or the SOTA detection networks on the DeepLesion dataset. This is because we have used 3D information input, multi-scale feature extraction, and a joint attention mechanism that preserves location information to extract richer features from 3D CT slices. The above results prove the effectiveness of our proposed method.

### 5.4 Effectiveness of loss function

As shown in Table 6, we compare the results of our proposed VDIoU loss with commonly used loss functions such as smooth-L1 loss and several common IoU losses on mAP. The ordinate is the mAP, and the abscissa is the number of iterations. It can be seen that the method that we proposed can quickly improve the map result in the early stage of training. At this time, the map obtained by our method is higher than other methods, which proves that our loss function can speed up the initial training speed of the model. After reaching convergence, our loss function can also achieve the best results. This proves that the proposed loss function can speed up the model’s initial training speed and improve the accuracy after the final convergence.

**TABLE 6:** mAP results after different iterations of training with different loss function.

	Iteration						
	5000	10000	15000	20000	25000	30000	35000
Smooth-L1	12.7	18.8	22.5	26.4	29.3	32.6	34.1
IoU	9.8	15.7	21.1	25.0	31.4	32.8	35.3
DIoU	13.1	18.3	22.7	27.1	31.2	34.6	36.2
Ours	9.3	19.7	24.8	29.2	33.6	35.4	36.3

## 6 Discussion

In this section, we first summarize the main differences between the proposed FPN-based backbone model, PAC-Net, and previous studies on medical image detection. We also point out the limitations of our proposed model as well as potential solutions to deal with these limitations in the feature.

### 6.1 Comparison with previous work

Our proposed PAC-Net uses a position-guided attention module to solve the position offset problem caused by upsampling in FPN. Unlike the attention module of previous methods, we use feature maps with accurate position information to generate position weight matrices to guide high-level features with rich semantic information so that the fused features have both rich semantic information and accurate position information to enhance the comprehensive representation of features. Compared with the IoU-based loss functions commonly used in medical image detection, our proposed VDIoU loss also has some improvements. In some cases, the common IoU-based losses can degrade or even fail. Therefore, we differ from the common IoU-based losses by using the sum of the vertex distances of GT and RP divided by the diagonal distance of the minimum enclosing subsection as an additional penalty term of the IoU loss to avoid the above problem. At the same time, this computation process does not add too much computation and thus affects the training speed. Therefore, our proposed VDIoU loss can help the network converge more accurately and quickly. In addition, PAC-NeT also adds an additional SPP block to expand the feature map receptive field and thus enhance the overall feature characterization capability of the network.

### 6.2 Limitations and future work

Although our proposed PAC-Net achieves good performance in our task, its performance can be further improved in the future by carefully addressing the following limitations or challenges.

First, in our current implementation, the fusion of FPN features at different scales results in information loss. FPN loses semantic information during the fusion of deep and shallow features as the number of channels is changed by 1 × 1 convolution to make them fuse with each other, thus weakening the expressiveness of multi-scale features. Therefore, it is a future research direction to find a more efficient feature fusion method to minimize the loss of semantic information during the fusion process of features at different scales. Second, insufficient data for medical image detection has been a challenge, where the annotation is time-consuming and requires the collaboration of researchers and radiologists. We plan to expand the amount of data and thus the capability of the model by some data augmentation methods in the future.

## 7 Conclusion

In this paper, we first identified the existing shortcomings of FPN-based medical lesion detection models and then proposed a novel FPN-based backbone model, PAC-Net, to remedy these problems and achieve better medical lesion detection. We conducted extensive experiments and the results demonstrate that i) the proposed PAC-Net achieves a better detection accuracy than the state-of-the-art baseline. ii) The advanced modules, PAC and VDIoU, are both effective and important for the PAC-Net to achieve a superior lesion detection performance. In the future, we intend to apply PAC-Net to more practical medical imaging lesion detection tasks to validate its applicability and scalability.

## Data Availability

Publicly available datasets were analyzed in this study. This data can be found here: https://nihcc.box.com/v/DeepLesion.

## Footnotes

1
https://nihcc.app.box.com/v/DeepLesion.
